# Considerations on the use of microsensors to profile dissolved H_2_ concentrations in microbial electrochemical reactors

**DOI:** 10.1371/journal.pone.0293734

**Published:** 2024-01-19

**Authors:** Tobias Sandfeld, Louise Vinther Grøn, Laura Munoz, Rikke Louise Meyer, Klaus Koren, Jo Philips

**Affiliations:** 1 Department of Biology, Aarhus University, Aarhus, Denmark; 2 Department of Biological and Chemical Engineering, Aarhus University, Aarhus, Denmark; 3 Interdisciplinary Nanoscience Center, Aarhus University, Aarhus, Denmark; Siksha O Anusandhan University Institute of Technical Education and Research, INDIA

## Abstract

Measuring the distribution and dynamics of H_2_ in microbial electrochemical reactors is valuable to gain insights into the processes behind novel bioelectrochemical technologies, such as microbial electrosynthesis. Here, a microsensor method to measure and profile dissolved H_2_ concentrations in standard H-cell reactors is described. Graphite cathodes were oriented horizontally to enable the use of a motorized microprofiling system and a stereomicroscope was used to place the H_2_ microsensor precisely on the cathode surface. Profiling was performed towards the gas-liquid interface, while preserving the electric connections and flushing the headspace (to maintain anoxic conditions) and under strict temperature control (to overcome the temperature sensitivity of the microsensors). This method was tested by profiling six reactors, with and without inoculation of the acetogen *Sporomusa ovata*, at three different time points. H_2_ accumulated over time in the abiotic controls, while *S*. *ovata* maintained low H_2_ concentrations throughout the liquid phase (< 4 μM) during the whole experimental period. These results demonstrate that this setup generated insightful H_2_ profiles. However, various limitations of this microsensor method were identified, as headspace flushing lowered the dissolved H_2_ concentrations over time. Moreover, microsensors can likely not accurately measure H_2_ in the immediate vicinity of the solid cathode, because the solids cathode surface obstructs H_2_ diffusion into the microsensor. Finally, the reactors had to be discarded after microsensor profiling. Interested users should bear these considerations in mind when applying microsensors to characterize microbial electrochemical reactors.

## Introduction

Microbial electrochemical processes, such as microbial electrosynthesis (MES), are of high interest to develop biotechnologies for the utilization of CO_2_ and the storage of renewable electric energy [1–3]. MES relies on microorganisms, such as acetogenic bacteria or methanogenic archaea, capable of withdrawing electrons from a cathode to reduce CO_2_ into more valuable products (i.e. organic acids, alcohols or methane) [4, 5]. Recent evidence has shown that H_2_ acts as a crucial intermediate in the cathodic electron uptake by these microorganisms [6–8], since the cathode electrochemically generates H_2_, which is rapidly consumed by the microorganisms. The acetogen *Sporomusa ovata*, for instance, has been described as the strain with the best performance for MES [9–11], while this attribute is possibly related to its specific H_2_ utilization characteristics [12, 13]. Measuring the distribution and dynamics of H_2_ in microbial electrochemical reactors is thus of importance to gain insights into the mechanisms behind microbial electrochemical processes. Standard gas chromatography (GC), however, only allows the analysis of gaseous samples, e.g. samples of the headspace or the off-gas of bioelectrochemical reactors. Nevertheless, the most interesting H_2_ concentrations are not those in the gas phase, but rather those in the liquid phase and in close proximity of the cathode surface, where the electrochemical and microbial reactions occur simultaneously.

Clark-type H_2_ microsensors provide the possibility to measure dissolved H_2_ concentrations, while their narrow tip allows to resolve the H_2_ distribution on a micrometer scale. Together with a micromanipulator system, microsensors can thus profile dissolved H_2_ concentrations with a micrometer-scale resolution. Moreover, H_2_ microsensors are sensitive (detection limit generally < 1 μM), fast responding (response time of a few seconds), highly accurate and almost non-invasive [14, 15]. Microsensors for H_2_ have been used for many years to study different natural environments on a microscale [16, 17]. Since bioelectrochemical and other biotechnologies are nowadays intensively being studied, H_2_ microsensors are also being used to obtain insights into engineered systems [8, 18–22]. Some studies have used H_2_ microsensors to measure H_2_ concentration changes over time in bioelectrochemical reactors (fixed distance from the cathode) [8, 21], while others already applied H_2_ microsensors to measure H_2_ profiles and investigate the H_2_ gradients towards a cathode (Table 1). None of these studies described their microsensor methodology in detail, except for a recent study that developed a method to apply microsensors in a specific electrochemical reactor (not standard reactor type, no microbes added) to profile through multiple porous cathodes [23]. Nevertheless, several complications arise when integrating microsensor profiling with the operation of even standard microbial electrochemical reactors.

**Table 1:** Comparison of studies that previously performed H_2_ microsensor profiling in microbial electrochemical reactor setups.

Reference	Microbial electrochemical reactor setup	Operation H_2_ microsensor profiling	Main trends observed from H_2_ concentration profile
Liu et al.,[24]	**Electrode**: Coated carbon cloth (porous) **Inoculum**: Methanogenic community	**Tip diameter**: 10 μm No information given on **resolution** or **flushing** Reactor was 90° rotated before profiling to orient electrode horizontally Profiling performed through porous electrode	H_2_ profiles measured at different time points: Gradual increase towards cathode at one time point
Kracke et al., [20]	**Electrode**: Solid, custom-made material **Inoculum**: *Methanococcus maripaludis*	**Tip diameter**: 20–40 μm **Resolution**: 50–500 μm **Headspace flushing**: N_2_/CO_2_ Profiled towards electrode Profiling stopped when change in sensor signal was observed, which was assumed to indicate that the sensor touched the electrode	**Abiotic reactors**: 220 μM H_2_ throughout liquid phase **Biotic reactors**: 0.2–0.6 μM H_2_ throughout liquid phase, with sharp increase towards cathode surface (< 100 μm)
Cai et al., [22]	**Electrode**: Treated carbon cloth (porous) **Inoculum**: Methanogenic community	**Tip diameter**: 10 μm **Resolution**: 100 μm **Headspace flushing**: N_2_ Profiled towards electrode, until electrode moved	Smooth increase towards cathode surface
de Smit et al., [23]	**Electrode**: Three layers of carbon felt (porous) Almost horizontal orientation (17°) **Inoculum**: none	**Tip diameter**: 50 μm **Resolution**: 100 μm **Gas sparging**: N_2_/CO_2_ Reactor shortly opened to replace cap with custom-made sleeve for leak-free movement of the microsensor Profiling performed through porous electrodes	Highest H_2_ levels inside of the cathode layer closest to the membrane Low H_2_ levels in part of catholyte due to gas sparging No H_2_ with unconnected electrode
Boto et al. [25]	**Electrode**: Graphite (solid) Horizontal orientation **Inoculum**: *Clostridium ljungdahlii*	No information given on **tip diameter** and **resolution** No **headspace flushing**, reactor opened in customized anaerobic box No spinning during profiling	Gradual increase towards the cathode surface Lower H_2_ concentration on cathode with biofilm- dominant strain versus abiotic control
This study	**Electrode**: Graphite (solid) Horizontal orientation **Inoculum**: *Sporomusa ovata*	**Tip diameter**: 25 μm **Resolution**: 25–100 μm **Headspace flushing**: N_2_/CO_2_ Microsensor placed on cathode surface by aid of stereomicroscope Profiles measured towards headspace	Accumulation of H_2_ increasing over time in abiotic reactors H_2_ levels maintained low over time in biotic reactors Similar concentrations throughout liquid phase, but often peak observed at a short distance from cathode

In this study, we developed a microsensor method to measure and profile the dissolved H_2_ concentration in commonly used H-cell type bioelectrochemical reactors. This work elaborates on all the considerations that were taken into account to reconcile microsensor measurements with bioelectrochemical reactor operation. We tested our microsensor method by recording a time series of H_2_ profiles in three sets of H-cell reactors with or without inoculation of *S*. *ovata*. We also compared microsensor measurements of the dissolved H_2_ concentration at the gas-liquid interface, with headspace GC measurements. Our results demonstrate that our microsensor method provides insightful H_2_ profiles with a high resolution. Nevertheless, we also identified various important limitations of the use of H_2_ microsensors in microbial electrochemical systems.

## Material and methods

### 1. Microbial electrochemical reactors

**Fig 1:**
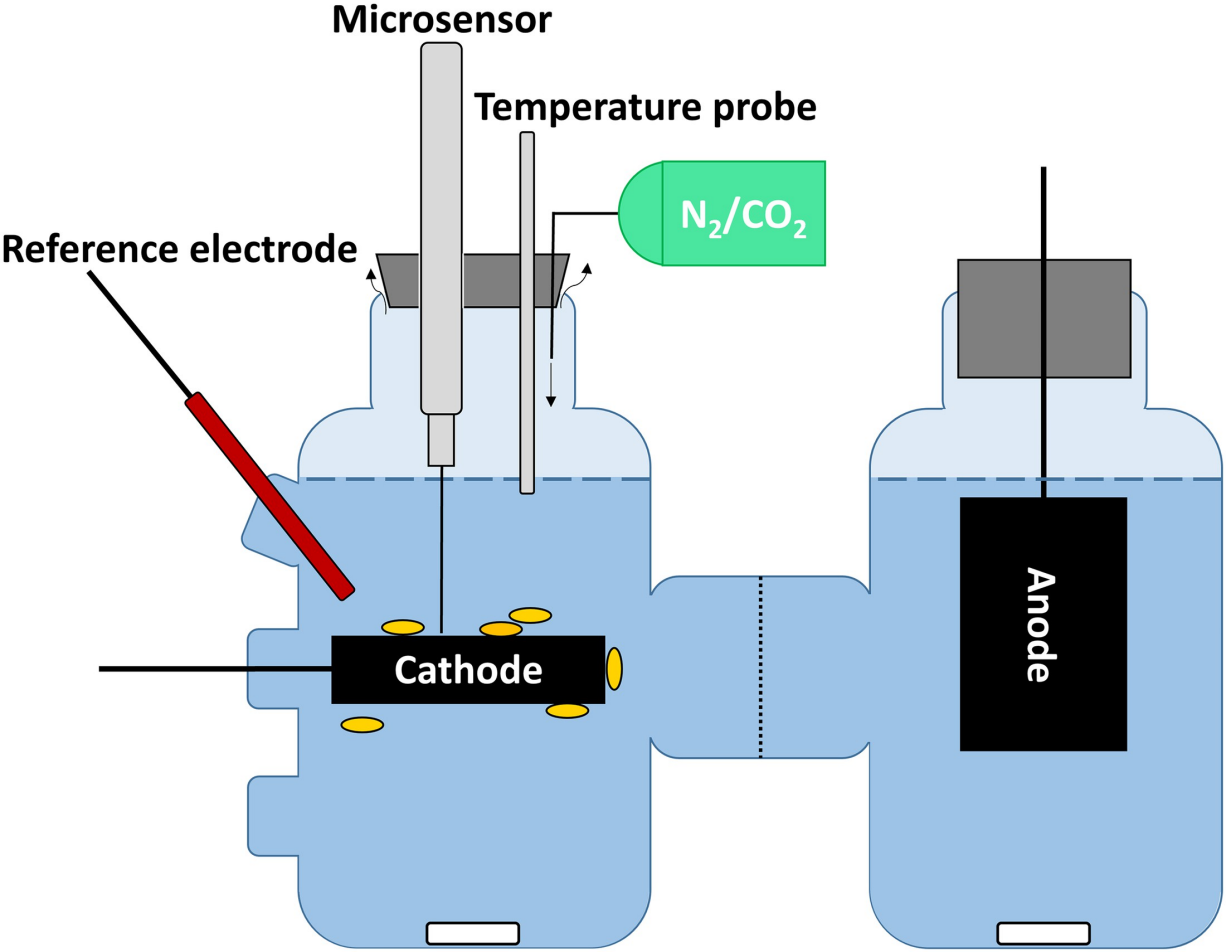
Illustration of the H_2_ microsensor set-up in the microbial electrochemical reactor. The cathode was placed horizontally, enabling the use of micromanipulator and profiling from the cathode surface towards the gas-liquid interface (indicated by the dark blue dashed line). The temperature probe was placed in the medium and a heating plate maintained a medium temperature of 30°C. The headspace was flushed with N_2_/CO_2_ gas. The rubber stopper closing the cathode chamber was not gastight, as indicated by the curved arrows, and hence gas could leave the chamber. *Sporomusa ovata* (yellow shapes) had been inoculated in the cathode chamber. The anode was placed vertically. A proton exchange membrane separated the cathode and anode chambers. The cathode, anode and reference electrode were connected to a potentiostat, poising the cathode at -610 mV vs. SHE, also during the H_2_ microsensor measurement. The medium was mixed using a magnetic stirring bar, also during microsensor profiling.

**Fig 2:**
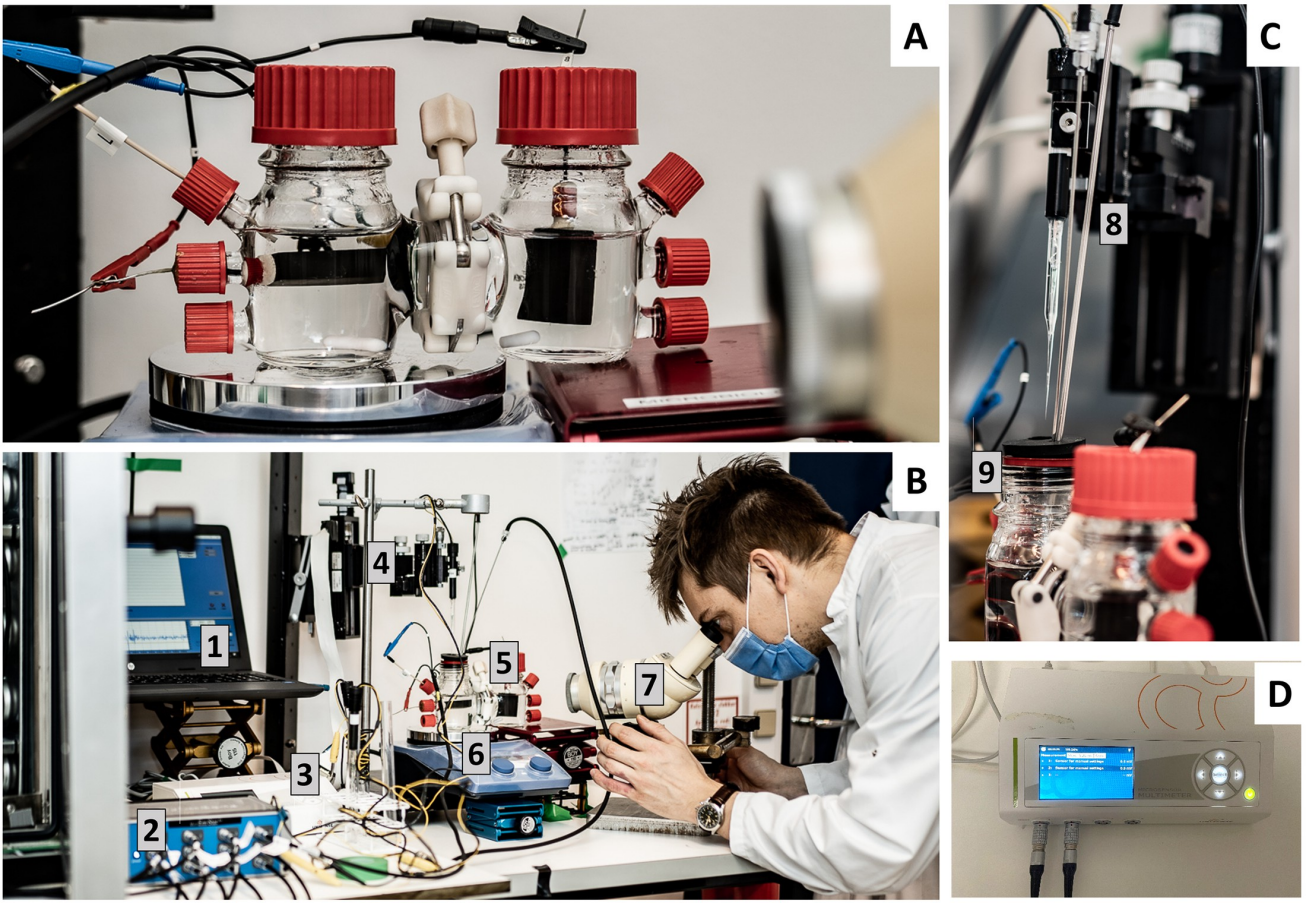
Images of the bioelectrochemical reactor and the bench-top H_2_ microsensor measurement set-up. **A**: The bioelectrochemical reactor before the system was opened, with the cathode placed horizontally. **B**: H_2_ microsensor set-up in practice: **1)** Computer used for data acquisition and to control the microprofiling system; **2)** Potentiostat controlling the potential of the cathode; **3)** Microsensor multimeter with H_2_ microsensors connected (also shown in D); **4)** Micromanipulator system used for μm control of the H_2_ microsensors in the bioelectrochemical reactor; **5)** H-cell with a three-hole rubber stopper closing the cathode chamber, making the chamber accessible for the microsensor, temperature probe and the gas inlet; **6)** Heating plate with temperature control and stirring; **7)** Stereo microscope used for placing the H_2_ microsensor tip precisely on the cathode surface. **C**: **8)** H_2_ microsensor mounted on the micromanipulator system and ready for profiling. **9)** Three-hole rubber stopper with the microsensor, temperature probe and the gas inlet in use. **D**: Multimeter connected to the H_2_ microsensors. Photo credit: Kasper Hornbæk. The individual shown in this figure has given written informed consent (as outlined in PLOS consent form) to publish this picture.

The bioelectrochemical reactors used in this study were standard H-cell type reactors (Fig 1), constructed similarly to Yee et al. [26]. Reactors consisted of two glass chambers (Adam & Chittenden, USA), with GL45 top ports and three GL12 side ports (Figs 1 and 2A). Both the working and counter electrode (cathode and anode, respectively) were constructed from sanded (P240) carbon graphite blocks (35x25x10 mm, EDM-3 Novotec), cleaned with ultrasonication, followed by an acid (1 M HCl) and base (1 M NaOH) wash, and rinsed with MilliQ water. A titanium wire (1 mm, 99.99%, Alfa Aesar) was connected to the carbon graphite in a drilled hole, which was kept in place with conductive silver epoxy (Electrodag, 5810) and screw fittings with the openings covered by vacuum epoxy (Torr Seal, TS10). The anode was pierced through the butyl rubber stopper of the top port (vertical orientation) and the cathode through a butyl rubber seal of the side port (horizontal orientation) (Figs 1 and 2A). The two reactor chambers were separated by a proton exchange membrane (N117, Nafion) (Fig 1). A Ag/AgCl 3.4 M KCl reference electrode (LF-2-100, Innovative Instruments, USA, +205 mV vs. SHE) was included in the cathode chamber (Figs 1 and 2A). Each chamber was filled with 100 mL anoxic medium containing: 0.5 g NH_4_Cl, 0.5 g MgSO_4_ x 7 H_2_O, 0.25 g CaCl_2_ x 2 H_2_O, 2.25 g NaCl, 2 mL FeSO_4_ x 7 H_2_O solution (0.1% in 0.1N H_2_SO_4_), 0.1 g yeast extract, 0.35 g K_2_HPO_4_, 0.23 g KH_2_PO_4_, 4 g NaHCO_3_, 1 mL trace element solution [27], 1 mL selenite-tungstate solution [27] and 10 mL Wolin’s vitamin solution (DSMZ 141) per liter and with 50 mM 3-(N-morpholino)propanesulfonic acid (MOPS, brought to pH 7.2 with NaOH). Both reactor chambers were stirred with magnetic stirring bars (Figs 1 and 2A).

### 2. Sporomusa ovata cultivation and reactor inoculation

The acetogenic bacterium *Sporomusa ovata* DSM-2663 was revived from a frozen stock of our culture collection in anoxic medium adjusted to heterotrophic growth. The composition of this medium was similar to the one described for the reactors above, but without MOPS, with more yeast extract (2 g·L^-1^), and with the addition of betaine x H_2_O (6 g·L^-1^) and L-cysteine-HCl x H_2_O (0.3 g·L^-1^). *S*. *ovata* was transferred once to 100 mL of the same medium (5% inoculation). Cells in the exponential growth phase (OD_600_ at 0.5) were harvested, spun down, and washed twice before suspension in the medium used in the reactors (described above). Cathode chambers with 100 mL anoxic medium were inoculated with 1 mL washed cell concentrate for a start OD_600_ of 0.1. The reactors were flushed for about 30 min with N_2_/CO_2_ gas (80:20) after inoculation to remove potential traces of atmospheric oxygen, and subsequently pressurized to a total pressure of 1.1 bar at room temperature. The reactors were placed in a 30 °C incubator and connected to a potentiostat (MultiEmStat+, PalmSens, Netherlands) with the cathode poised at -610 mV vs. SHE (Fig 2B 2). For the test experiment, six reactors were built, of which three were inoculated and three were used as abiotic controls. After 3 hours (day 0), 5 days and 17 days, one biotic and one abiotic reactor, in pairs, were used for H_2_ microsensor measurements, after which they were discarded.

### 3. Preparation of the reactors for microsensor measurements

Microsensor measurements were initiated by carefully taking the reactors out of the incubator, while keeping them connected to the potentiostat (Fig 2B). The cathode chamber was placed on a magnetic stirring plate (180 rpm), which was also equipped with a heating and a temperature probe for temperature control (Fig 2B 5 and 2B 6). The top rubber stopper of the cathode chamber was carefully opened at one side to start the flushing of the headspace with N_2_/CO_2_ gas. The flushing gas was preheated by flowing through metal tubing placed in a 32°C water bath. While flushing, the top rubber stopper was replaced with a rubber stopper with three holes. One hole for the microsensor, one for the temperature probe, and one for the flushing gas needle (Figs 1 and 2C 9). The temperature probe was placed in the catholyte and controlled the temperature of the liquid phase at 30°C during the measurements. Magnetic stirring continued during microsensor profiling.

### 4. H_2_ profiling using microsensors

Hydrogen microsensors (Unisense A/S, Denmark) with ~25 μm tip diameter were used for recording H_2_ profiles (Fig 2C 8). The sensors were connected to a four-channel multimeter with a built-in 16-bit A/D converter (Unisense Microsensor Multimeter, Ver 2.01; Unisense A/S, Denmark) (Fig 2B 3 & 2D). The sensors were calibrated according to the manufacturer´s guidelines. In short, pre-polarized sensors were calibrated prior to each profile measurement by a five-point-calibration (three measurements in each point). The calibration solutions were at 30°C and had dissolved H_2_ concentrations ranging from 0 to 38 μM, obtained by dispensing aliquots of saturated H_2_ stock solution (MilliQ bubbled with pure H_2_ gas) in medium. The detection limit was calculated as *3·SD/slope*, and the quantification limit as *9·SD/slope* [28], with *SD* being the standard deviation of the microsensor signal measured three times in milliQ water (blank measurements), and *slope* the slope of the calibration curve.

The H_2_ microsensor was mounted on a motorized micromanipulator system (Unisense A/S, Denmark) (Fig 2B 4 and 2C 8). Data acquisition and control of the micromanipulator system were enabled with the software program SensorTrace PRO (Unisense A/S, Denmark) **(**Fig 2B 1). To start the profiling, the tip of the microsensor was positioned above the middle of the cathode surface and carefully lowered by manually handling the screw of the micromanipulator, until the tip was placed exactly on the cathode surface, as observed with a stereomicroscope (Fig 2B 7). Afterwards, the micromanipulator moved the tip of the microsensor with μm-scale steps upwards, towards the liquid-gas interface. In the first 1000 μm, the dissolved H_2_ concentration was recorded with 25 μm steps, while H_2_ was measured each 100 μm between 1000 and 5000 μm distance from the cathode. The measurement of one profile took in total around 20 min (6 seconds waiting time and 1 second measuring time at each depth). All reactors were discarded after profiling.

### 5. Measurement of headspace H_2_ with GC

The H_2_ partial pressure in the headspace of the reactors incubated for 17 days was measured using GC. Prior to microsensor profiling, the total pressure of the headspace was measured with a digital manometer and 1.5 mL gas was sampled from the headspace using a syringe. This procedure was conducted immediately before the reactors were opened and the N_2_/CO_2_ flushing of the headspace was started to initiate microsensor profiling. Headspace samples were analyzed using a CompactGC (Interscience, Netherlands), as previous described [12]. The measured H_2_ value in the headspace (obtained in ppm) was converted to the H_2_ partial pressure (Pa) using the total pressure. The corresponding dissolved H_2_ concentration in the liquid phase was calculated using the Henry’s law constant (7.8·10^−4^ M·atm^-1^, at 30°C) [29]. Expressed as a dissolved H_2_ concentration, our GC method has a detection limit of 0.004 μM.

## Results and discussion

### 1. Set-up requirements for H_2_ microsensor profiling in microbial electrochemical systems

Microsensor profiling with a high resolution requires the use of a motorized micromanipulator system. Such a system can only optimally move a microsensor in a vertical direction. For this reason, we placed the cathode horizontally in standard H-cell type reactors (Fig 1), instead of the common vertical orientation. Similarly, Liu et al. [24] and Veerubhotla and Marzocchi [30] rotated their whole reactor to enable microsensor measurements, while Boto et al. [25] also placed their electrodes horizontally (Table 1). In addition, a horizontal cathode orientation enables the measurement of vertical H_2_ profiles from the cathode surface towards the gas-liquid interface of the bioelectrochemical reactors. Nevertheless, a horizontal cathode orientation could affect the electrochemical processes occurring in the reactors, for instance because of the different position of the cathode versus the membrane and anode or the altered mixing of the catholyte. Fortunately, we did not observe an impact of this horizontal orientation, since a test experiment gave comparable current consumptions with vertical and horizontal oriented cathodes, and no significant difference in the rate of H_2_ accumulation in the headspace was observed (Fig B in S1 Appendix). Furthermore, we always placed the microsensors at the middle of the cathode, to exclude a possible effect of the distance from the membrane or of the sides of the electrode on the H_2_ concentration profile.

Another requirement is that the microsensor tip is placed exactly on the cathode surface, to precisely set the zero of the measurements. The microsensors’ needle shaped measuring tip, however, is made from glass capillaries with diameters in the low end of the micrometer scale, making them extremely fragile. Thus, measurements near or on solid cathodes entail a high risk to break the microsensors. Others have used porous electrode materials, allowing the sensor to pierce through the electrode [23, 24]; or have profiled towards the electrode until a change in the sensor signal was observed [20] (Table 1). Here, we used a stereomicroscope to precisely place the microsensor on the cathode surface and measured the profile in the direction away from the cathode. However, even this procedure could not completely prevent all breakage of microsensors. Fortunately, H_2_ microsensors are relatively cheap and easy to replace.

To conduct the H_2_ microsensor profiling in H-cells, it is necessary to open the reactors. However, microbial electrochemical processes often rely on anaerobic bacteria, such as *S*. *ovata*. It is thus important to maintain anoxic conditions during the profiling and avoid the disturbance of the microbial and the electrochemical processes by the entrance of oxygen. Several studies previously used headspace flushing to maintain the anoxic environment [20, 22], while Boto et al. [25] opened their reactors in a customized anaerobic box (Table 1). Here, we also flushed the headspace continuously during the microsensor measurements. Consequently, the headspace atmosphere, containing some H_2_ before the start of the measurements, was exchanged during the profiling. It should be noted that extensive headspace flushing affected the H_2_ concentrations throughout the reactor, as discussed below.

Our reactors were incubated in a closed incubator at 30°C to meet *S*. *ovata*’s temperature requirements. For our setup, it was practically impossible to perform the H_2_ microsensor measurements with the microprofiling system inside the incubator, as would presumably also be the case for setups in many other labs. Thus, it was necessary to take the reactors out of the incubator for profiling. To minimize the disturbance of the electrochemical and microbial processes, while profiling the reactor outside of the incubator, the reactor needs to remain connected to the potentiostat and its temperature should be controlled. In addition, temperature control is crucial to obtain stable microsensor readings, as temperature influences the microsensors’ signal (expected sensitivity of about 1–3% per °C according to manufacturer). Hence, temperature gradients in the liquid phase, created in case the reactors would be moved from 30°C to room temperature, could alter the response of the H_2_ microsensor and lead to erroneous H_2_ profiles. None of the previous studies using H_2_ microsensors in microbial electrochemical reactors mentioned how they incorporated temperature control during profiling (Table 1). Here, we strictly controlled the temperature of the liquid phase of the reactors (using a temperature probe and heating plate), as well as that of the headspace (using preheated gas), to 30°C during profiling.

Microsensor measurements further require selection of the most appropriate tip diameter, as this property defines the highest obtainable spatial resolution of the measurements [14]. Previously, tip diameters ranging from 10 to 50 μm and resolutions ranging from 50 to 500 μm have been used (Table 1). In our case, we expected that the interesting changes in H_2_ concentration would occur within the first couple of hundred micrometers from the cathode surface, as was suggested by the results of Cai et al. [22] and Kracke et al. [20] (Table 1). Therefore, we chose a H_2_ microsensor with a 25 μm tip diameter, which enabled measurements with a 25 μm resolution. A finer resolution could be obtained by selecting a microsensor with an even narrower tip. H_2_ microsensors with tip diameters down to 10 μm are commercially available (Unisense A/S, Denmark), while tip diameters down to 2 μm are reported in the literature [31, 32]. However, this compromises the sensitivity of microsensor, since the detection limit of a microsensor inversely relates to its tip diameter [33], as further discussed below.

### 2. H_2_ profiles: An example of microsensor measurements in microbial electrochemical reactors

**Fig 3:**
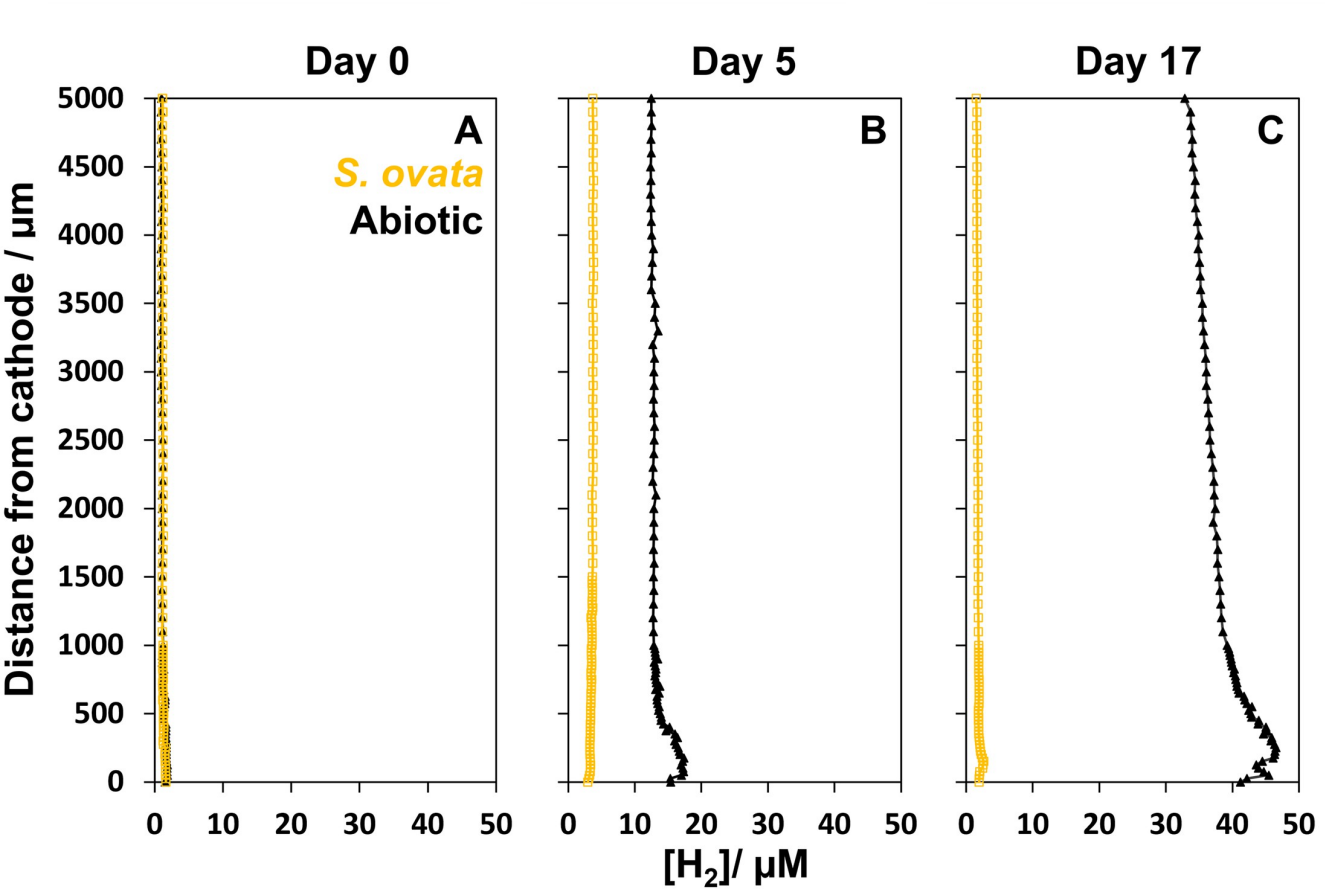
Microsensor profiles measured in abiotic and biotic reactors. Dissolved H_2_ concentration profiles were measured from the cathode surface (distance 0 μm) till 5000 μm towards the gas-liquid interface in bioelectrochemical reactors incubated with *Sporomusa ovata* (yellow squares) and without inoculum (black triangles) at day 0 (**A**), day 5 (**B**), and day 17 (**C**). The y-axis displays the distance from the cathode surface in μm, and the x-axis shows the dissolved H_2_ concentration in μM. The profiles were measured at three time points (day 0, day 5 and day 17) each in a different reactor, in total six reactors. Each reactor was discarded after profiling.

To test the developed microsensor method, H_2_ profiles were measured in H-cells inoculated with *S*. *ovata* and abiotic controls. Microsensor measurements were conducted on both an abiotic and a biotic reactor after 3 hours (day 0), 5 days and 17 days of incubation (one replicate at each time point) (Fig 3). As an example, the current consumption for the reactors incubated for 17 days is shown in Fig A in S1 Appendix. The microsensor measurements showed that H_2_ production began immediately after the start of the reactors. Already after 3 hours of incubation, H_2_ had accumulated to measurable levels above the cathode surface and a H_2_ gradient could be detected in both the inoculated and abiotic H-cell, even though the H_2_ concentration was below 2 μM in both reactors (Fig 3A). After 5 days of incubation, the H_2_ concentration in the reactor with *S*. *ovata* was about 4 μM throughout the liquid phase, whereas the abiotic reactor had a higher H_2_ concentration throughout the liquid phase (13 μM) and showed an apparent H_2_ peak of 17 μM at about 75 μm from the cathode surface (Fig 3B). A similar pattern, but more pronounced, was recorded in the reactors incubated for 17 days. In the *S*. *ovata* reactor, the H_2_ concentration was 2 μM throughout the liquid phase with a minor peak of 3 μM at about 150 μm from the cathode, while the abiotic reactor again had a higher H_2_ concentration throughout the liquid phase (35 μM, slightly increasing towards the cathode) with an apparent H_2_ peak of 45 μM close the cathode surface (Fig 3C). Similarly, the microsensor measurements of Kracke et al. (2019) found that the methanogen *Methanococcus maripaludis* maintained low H_2_ levels throughout the liquid phase (0.2–0.6 μM), while H_2_ had accumulated in an abiotic control up till 220 μM (Table 1). Others rather recorded H_2_ concentration profiles gradually increasing towards the cathode surface (Table 1), which was possibly due to the lack of stirring during microsensor profiling [25].

**Fig 4:**
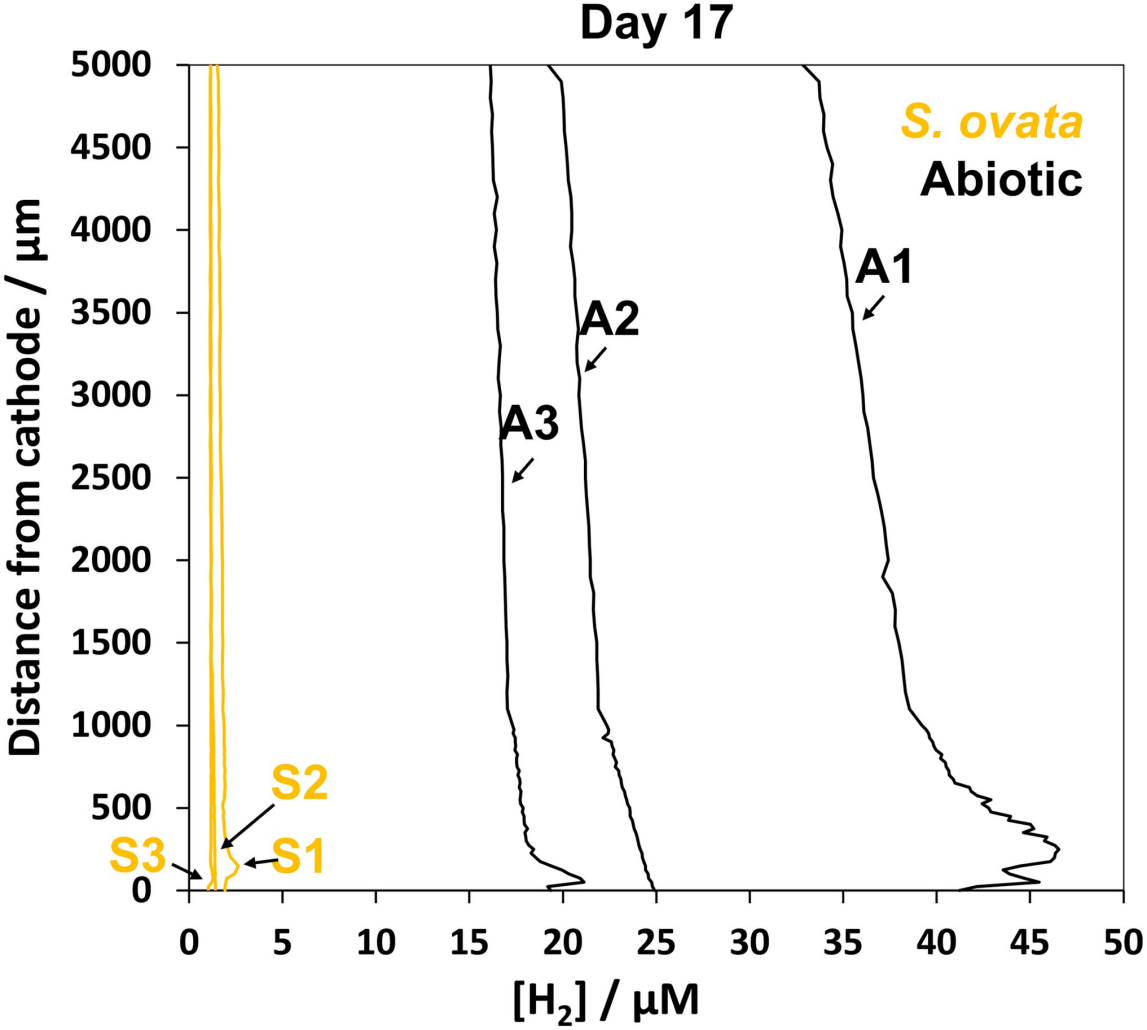
H_2_ profiles illustrating the drift in the dissolved H_2_ concentration due to headspace flushing. The y-axis displays the distance from the cathode surface in μm, and the x-axis shows the dissolved H_2_ concentration in μM. **S1**: first profile (t = 0 min) measured in the reactor with *Sporomusa ovata*, **S2**: second profile (t = +23 min), and **S3**: third profile (t = +45 min). **A1**: first profile (t = 0 min) measured in the abiotic reactor, **A2**: second profile (t = +22 min), and **A3**: third profile (t = +44 min).

Here, we confirmed the difference between the *S*. *ovata* and abiotic reactors by GC measurements on headspace samples taken just before the microsensor measurements performed at day 17 (Table A in S1 Appendix). The difference in the dissolved H_2_ concentration at the gas-liquid interface between the abiotic and inoculated reactor was though larger, when measured with GC, than when measured with microsensors. The headspace was sampled before opening of the reactors and the start of the headspace flushing, which could thus explain the variation between the H_2_ concentration determined with GC and microsensors. The headspace flushing likely stripped H_2_ from the liquid phase, which may have caused an underestimation of the H_2_ concentration measured by microsensors. We indeed observed that prolonged flushing of the headspace led to a drift of the H_2_ profiles, as became evident from repeated microsensor measurements on the same reactors (Fig 4). Profiles measured after 45 min of headspace flushing even showed that the dissolved H_2_ concentration throughout the liquid phase of the abiotic reactors had at least halved in comparison to profiles measured shortly after the start of the flushing. In another test experiment, we decreased the flow of the flushing gas and found that the dissolved H_2_ concentration corresponded well with the H_2_ concentration measured with GC (Table A in S1 Appendix). Consequently, limiting headspace flushing and keeping experimental procedures as short as possible, is important to obtain accurate H_2_ microsensor measurements. Nevertheless, opening of the reactors to allow microsensor profiling will often have an effect on the dissolved H_2_ concentrations, as is further discussed below.

Overall, our H_2_ microsensor measurements showed that the abiotic H-cells accumulated H_2_ and formed H_2_ peaks near the cathode over time, while in the *S*. *ovata* H-cells, the dissolved H_2_ concentration remained low (< 4 μM) across the three incubations (Fig 3). Such insights are highly relevant to gain a better understanding of the mechanisms of microbial electrochemical processes. *S*. *ovata* has previously been described as the acetogenic strain with the best performance for MES [9, 10], but it is not yet clear why this strain is so well adapted to take up cathodic electrons. Philips [13] proposed that *S*. *ovata* is possibly simply better at utilizing H_2_ at low levels, giving it an advantage in bioelectrochemical reactor systems in comparison to other strains. Our microsensor results confirm that *S*. *ovata* is capable of maintaining low dissolved H_2_ concentrations, also in the vicinity of the cathode where H_2_ generation occurs, as indicated by the abiotic H_2_ profiles. Initially, a direct extracellular electron uptake mechanism was proposed for *S*. *ovata*, since H_2_ levels were not detectable [4, 34]. The microsensor results obtained here indicate that the H_2_ concentrations in those studies might have been under the detection limit of their measuring method.

### 3. Limitations of H_2_ microsensor profiling

**Fig 5:**
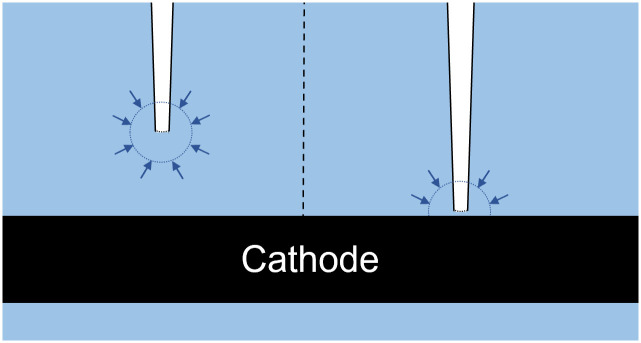
Illustration of the restriction diffusion volume in the near vicinity of the cathode surface. There is a spherical volume around the microsensor tip, supplying the microsensor with H_2_ by diffusion. Left shows the microsensor tip at a distance from the cathode, at which the diffusion sphere is not affected by the cathode. Right shows the microsensor tip in close proximity of the solid cathode, which blocks a part of the diffusion sphere and thereby restricts H_2_ diffusion into the microsensor. Ratios are not at scale. This scheme was inspired by an illustration of Unisense A/S.

In our H_2_ profiles, dissolved H_2_ concentrations often formed an apparent peak at a distance between 75 to 150 μm from the cathode surface (Figs 3B, 3C and 4). This peak was most clear in the profiles of the abiotic reactors, but was also observed in some profiles measured with *S*. *ovata*. This H_2_ peak gives the impression that the H_2_ concentration is higher at a certain distance from the cathode, than closer to the cathode surface (Figs 3 and 4). Nevertheless, as H_2_ is produced at the cathode surface, it is not expected that the H_2_ concentration would be lower very close to the cathode surface. A potential explanation is that the H_2_ concentration is underestimated when diffusion of H_2_ into the sensor becomes obstructed, by the cathode surface (Fig 5). The signal response of the microsensor is created by the diffusion of gas molecules (here H_2_) from a spherical volume around the sensor tip [33]. Thus, if the solid cathode blocks a part of this diffusion sphere, it hinders the diffusion of H_2_ into the microsensor. When the sensor tip is physically touching the cathode surface it is likewise expected that the diffusion path is even more obstructed (Fig 5). Hence, microsensors are likely incapable of accurately measuring H_2_ very close to the solid surface of a cathode. We expect that the distance till which microsensors can accurately measure H_2_ depends on their tip size, since microsensors with a smaller tip size have a smaller opening decreasing the diffusion path. We estimate that the distance from the solid surface needs to be at least twice the tip diameter, before measurements become accurate. Kracke et al. [20] also observed a change in microsensor signal, when approaching the cathode surface, and ceased profiling at this point (Table 1). Cai et al. [22] did not observe a H_2_ concentration peak, but profiled with a large resolution (100 μm) and small microsensor tip (10 μm) and used a porous electrode (Table 1), with which no diffusion restrictions are expected. Not all our profiles showed an apparent H_2_ peak above the cathode surface (Fig 4, A2). It is though important to realize that the absence of such an H_2_ peak does not exclude that the measured concentration on the cathode surface is underestimated due to diffusion limitation. Fig C in S1 Appendix illustrates that the effect of the diffusion restriction towards the cathode could be masked (no apparent H_2_ peak), in case of a steep incline of the H_2_ concentration towards the electrode.

We determined the detection limit of our H_2_ microsensors and found that this was 0.07 μM (±0.01) μM, while the quantification limit was 0.22 (±0.04) μM. These limits are in the same range as of standard GC methods (0.04% [35, 36]), corresponding to a dissolved H_2_ concentration of 0.3 μM). The GC-TCD method used in this study, which is configured to especially measure low H_2_ levels, has an order of magnitude lower detection limit (0.004 μM). Other specialized GC methods using a reducing compound photometer can even measure H_2_ down to 5 ppb, which corresponds to 4μ10^−6^ μM [37]. For our test experiment, the detection limit of the H_2_ microsensors was sufficiently low, since the lowest dissolved H_2_ concentration measured in our reactors (2 μM) was at least one order of magnitude higher than the detection and quantification limit (Fig 3). Nevertheless, in some cases, lower dissolved H_2_ concentration can be expected. For instance, we recently determined the H_2_ threshold, i.e. H_2_ concentration at which H_2_ consumption ceases, for *S*. *ovata* (6 Pa or 0.05 μM) [12], which is in the range of the detection limits of the microsensors applied in this work. Also some methanogens have H_2_ threshold in this range, while some sulfate reducers and other microbes even have lower H_2_ thresholds [38]. It is therefore important that interested users are aware that microsensors possibly are not capable of detecting and quantifying the lowest H_2_ levels that the microorganisms of their interest can create. Furthermore, we also recommend that H_2_ microsensors are calibrated with a range of low H_2_ concentrations, if accuracy in that range is of high importance, even though the microsensor signal is expected to be linear over its whole measurement range.

Biofilms with thicknesses up to 500 μm can form on the cathode surface [39], even though with *S*. *ovata* cathodic biofilms are usually rather thin (< 25 μm) [4]. In a related experiment, we found that *S*. *ovata* only formed a 4 μm thick cell layer, incompletely covering a horizontal cathode (Fig D in S1 Appendix). In case of thicker biofilms, it could be relevant to measure H_2_ profiles with microsensors in these cathodic biofilms to get insight into the transport processes occurring in those biofilms. The microsensors used in this study would not be capable of profiling cathodic biofilms thinner than 50 μm, since their tip diameter (25 μm) only allows for a 25 μm resolution. H_2_ microsensors with smaller tip diameters could be of interest to obtain a smaller spatial resolution. However, microsensors with such tiny tip diameters are less sensitive, as their small opening allows less gas to diffuse into the microsensor [33]. Consequently, H_2_ microsensors allowing a finer resolution, have higher detection limits, which could be above the H_2_ concentration in active MES systems, in which microbes maintain low H_2_ levels.

As described above, microsensor profiling requires to open microbial electrochemical reactors. Here, we avoided the intrusion of O_2_ by headspace flushing. Extensive headspace flushing, however, quickly stripped out H_2_ from the liquid phase (Fig 4). Opening the reactors in an anaerobic box, as by Boto et al. [25] could also prevent O_2_ intrusion, but is likely not always practically feasible. Alternatively, we found that without headspace flushing, O_2_ intrusion is rather slow, when the spinning of the liquid phase is turned off. However, this leads to very different concentration profiles than those in Fig 3, since a linear diffusion gradient between the cathode surface and the headspace would develop, as in [25]. Here, we conclude that minimizing the headspace flushing is the best option to avoid O_2_ intrusion (Table A in S1 Appendix). Unfortunately, none of these options completely prevent the loss of H_2_ from the systems, as the opening of the reactors will most likely cause a sudden drop of the headspace pressure and change in its composition. Ideally, microsensor profiling should be operated without opening of the reactors. De Smit et al. [23] used a custom-made sleeve for the leak-free movement of the microsensor, but still had to shortly opened their reactors to change one of reactor caps for this sleeve. Alternatively, microsensors placed in a needle are commercially available and can be pierced through a rubber stopper, but such a configuration cannot be used for profiling, as it cannot be handled by a micromanipulator.

A final limitation of the here described method is that the microbial electrochemical reactors were discarded after profiling. Liu et al. [24] did repeat microsensor profiling of the same reactor at different time points, but used a mixed culture inoculum (Table 1). Repeated profiling of the same pure culture reactor would be possible, if microsensors measurements could be performed under aseptic conditions. This could be attained by dipping the microsensors in ethanol and filter sterilizing the flushing gas, but would add additional complexity to the measuring procedure. In a test experiment, we applied aseptic conditions and continued the reactors after microsensor profiling. Despite our efforts to disturb the reactors only minimally during the whole procedure, we found that the current consumption profiles had irreversibly changed after the microsensor profiling (results not shown). For this reason, we chose to further use the microsensors only for endpoint measurements. The destructive nature of our method is, however, a clear drawback of the use microsensors in bioelectrochemical reactors.

## Conclusion

In this work, we described a H_2_ microsensor method to profile the dissolved H_2_ concentration in standard H-cell bioelectrochemical reactors. Our results demonstrated that this method provides insightful H_2_ profiles. Interested users should though be aware of the various limitations of the here described method. We found that headspace flushing (to maintain anoxic conditions during profiling) lowered the dissolved H_2_ concentrations over time. We thus conclude that keeping experimental procedures as short as possible, while minimizing headspace flushing, is crucial to obtain reliable H_2_ microsensor measurements. Furthermore, we discovered that the microsensors could not accurately measure H_2_ in the immediate vicinity of a solid cathode (distance less than twice the tip diameter). This limitation is likely due to restriction of H_2_ diffusion into the microsensor by the solid cathode surface. Bearing these limitations in mind, our microsensor setup and methodology can form a useful tool for scientists interested in measuring H_2_ in bioelectrochemical reactors. Moreover, the setup and restrictions described in this work are likely also relevant for other potentially useful microsensors (e.g. pH microsensors).

## Supporting information

S1 Appendix Supporting figs and table. (PDF)
