# Supplementary material for: Considerations on the use of microsensors to profile dissolved H2 concentrations in microbial electrochemical reactors
Source: PLoS One. 2024 Jan 19;19(1):e0293734. doi: 10.1371/journal.pone.0293734 (PMC10798470; doi:10.1371/journal.pone.0293734)
Supplement: S1 Appendix — (PDF) [file pone.0293734.s001.pdf]

S1 Appendix:
Supporting Figures and Table

Considerations on the use of microsensors to profile dissolved H_2 concentrations in microbial electrochemical reactors

Tobias Sandfeld[1¶], Louise Vinther Grøn[2¶], Laura Munoz[2], Rikke Louise Meyer[3], Klaus Koren[1], and Jo Philips[2*]

[1] Department of Biology, Aarhus University, Aarhus, Denmark

[2] Department of Biological and Chemical Engineering, Aarhus University, Aarhus, Denmark

[3] Interdisciplinary Nanoscience Center, Aarhus University, Aarhus, Denmark

*Corresponding author

email: jo.philips@bce.au.dk (JP)

¶ These authors contributed equally to this work.

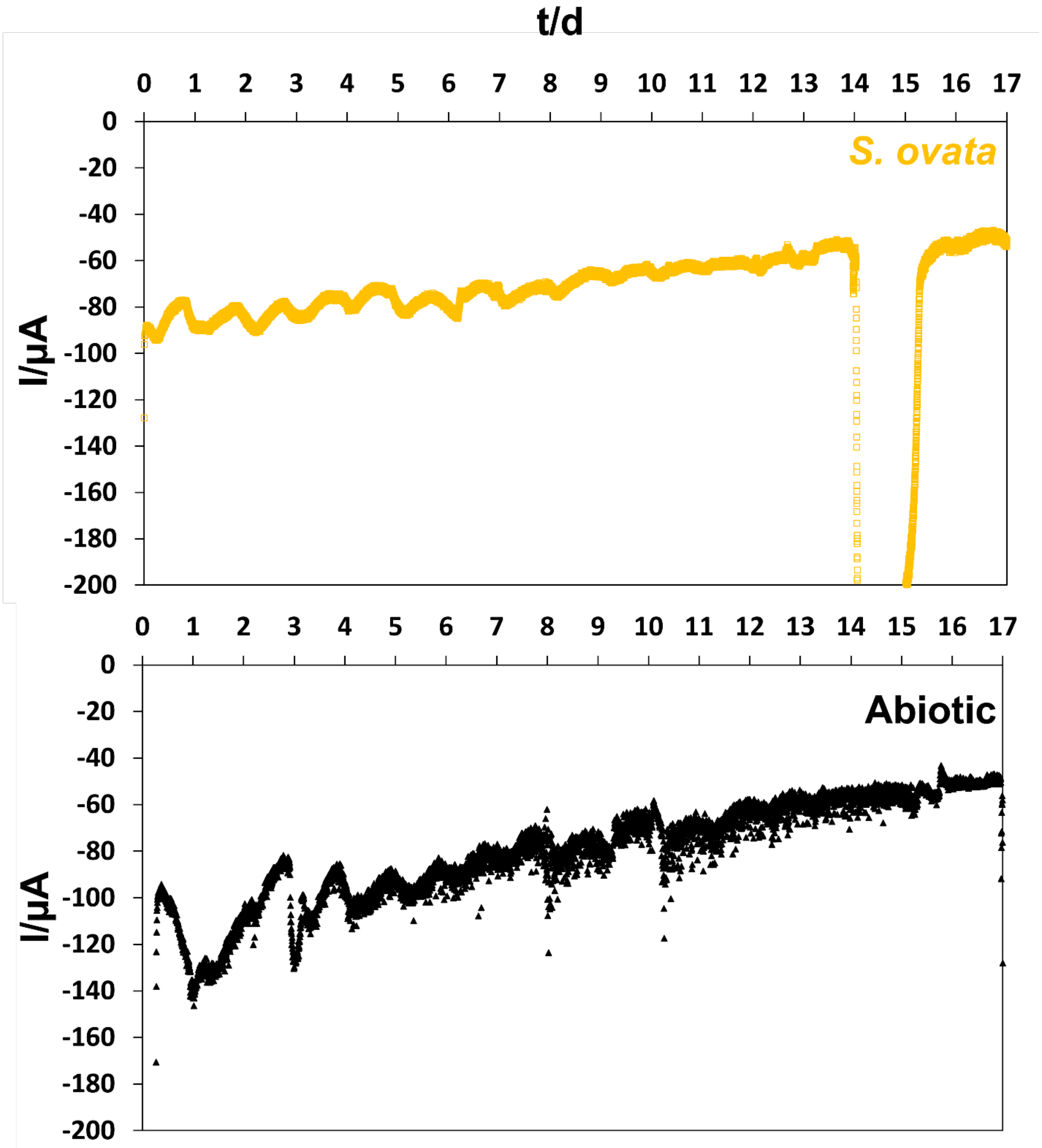

Fig A: Current consumption recorded for the reactor pair incubating for 17 days. The x-axis depicts the run time in days and the y-axis the current consumption in µA. Both reactors had a comparable current consumption. The large negative peak around 14 days for the reactor with *S. ovata* was due to a disturbance of the system.

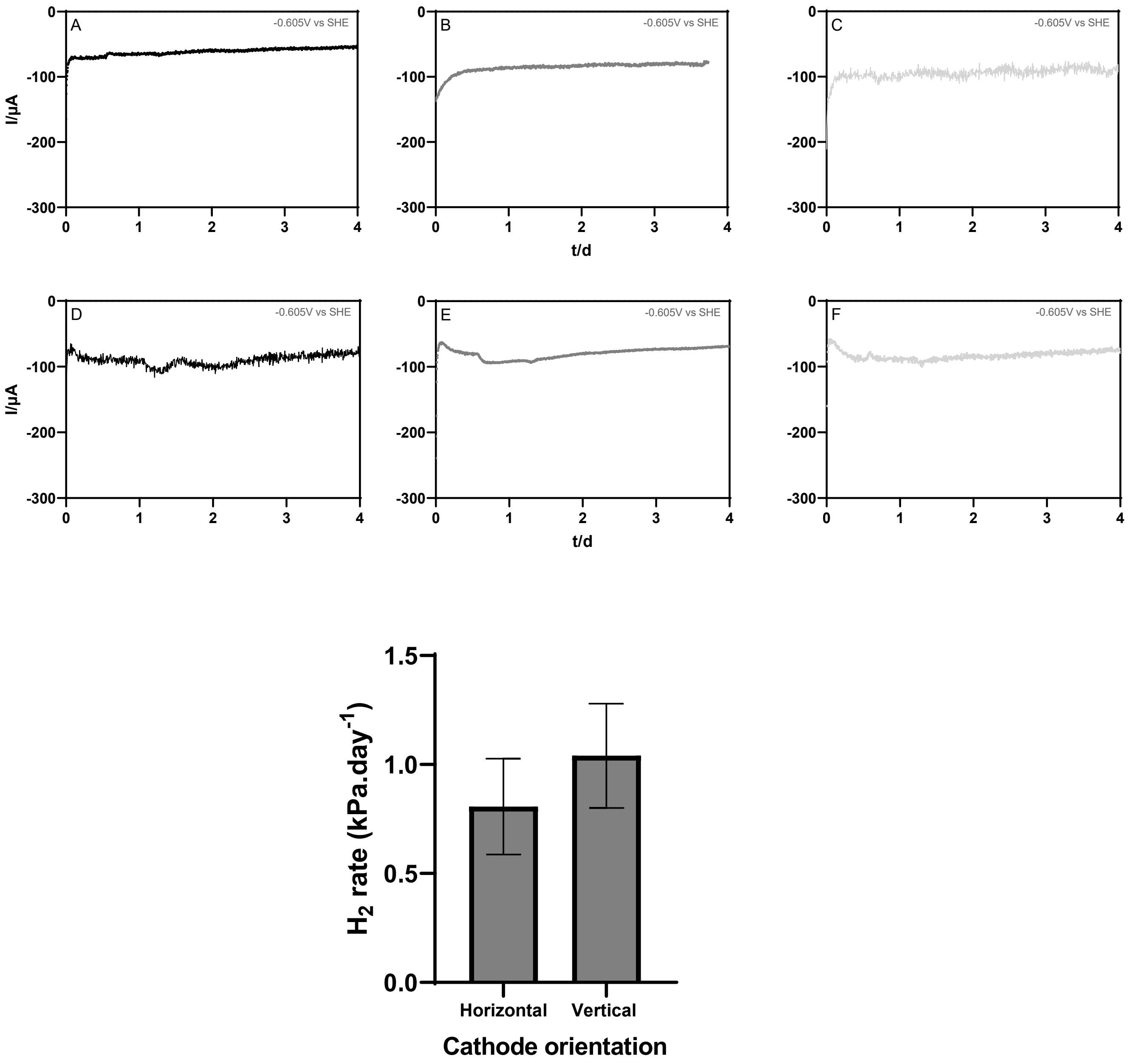

Fig B: **Top**: Current consumption over time in triplicated H-cell reactors with the cathode oriented horizontally (A, B, C) or vertically (D, E, F). All reactors were operated abiotically and cathodes were poised at a potential of -0.605 V vs. SHE. Comparable current consumptions were observed independent of the cathode orientation. **Bottom**: H_2 evolution rates over four days in the reactors with a horizontal versus vertical cathode (n = 3). The H_2 evolution rate was calculated from the accumulation of H_2 in the headspace, as measured with GC. No significant difference in the rate of H_2 accumulation was observed between the two cathode orientations.

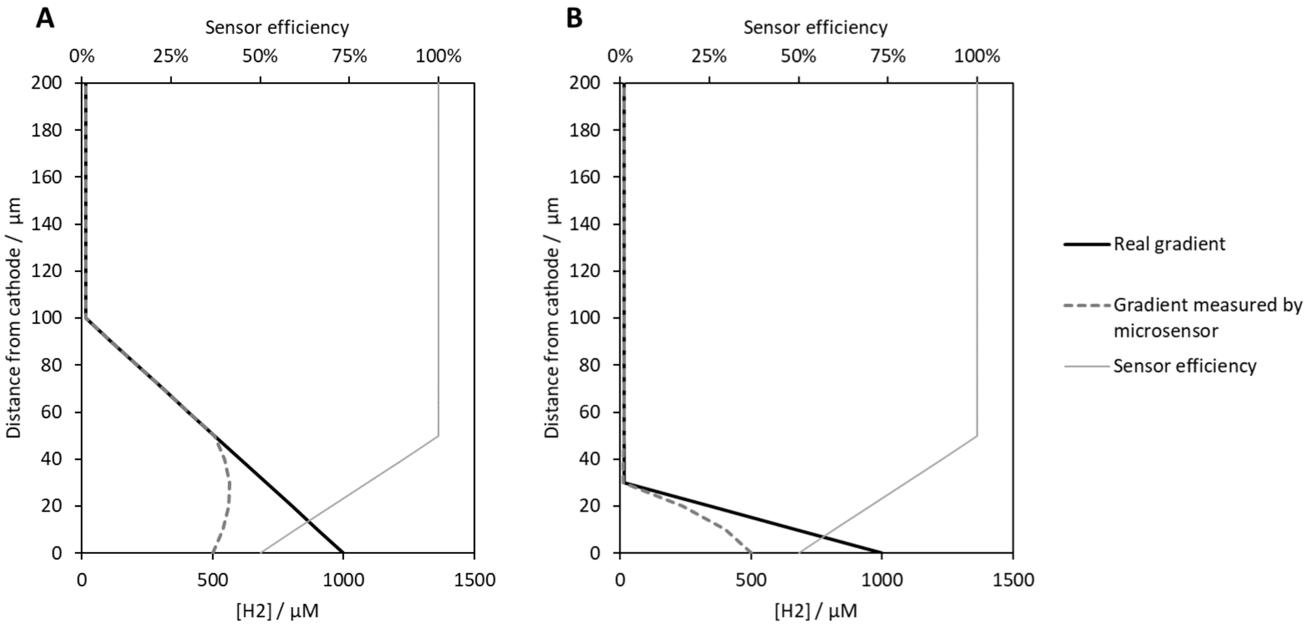

Fig C: Simulated effect of the underestimation of the dissolved H_2 concentration at close proximity of the solid cathode, due to restriction of H_2 diffusion into the microsensor. We assumed that the sensor signal would drop linearly over a distance of twice the microsensor tip size (50 µm) with 50%. The simulations show how the shape of the profile measured by the microsensor could have a H_2 peak (A) or no H_2 peak (B), depending on the real H_2 gradient.

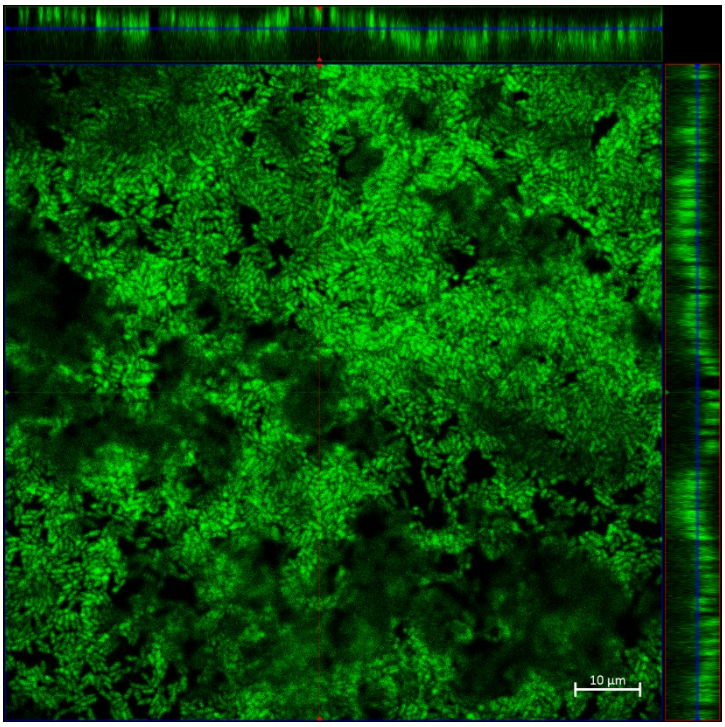

Fig D: Confocal laser scanning microscopy image of a *S. ovata* biofilm grown on a horizontal graphite cathode posed at -605 mV vs. SHE over a period of 13 days. The biofilm was fixed with 2.5% glutaraldehyde and stained with SYBRgreen II (at 20x concentration in PBS). The image was taken using a LSM700 confocal laser scanning microscope (Zeiss). The cells were imaged with a 63x, 1.4 NA oil immersion objective, excitation at 488 nm, and emission detected at 490-800 nm. The scale bar represents 10 µm. The Z-stack was 8 µm thick, and the image shows a maximum intensity projection with side view of a single position.

Table A: Comparison of measurements with two different methods of the dissolved H_2 concentration at the gas-liquid interphase of the reactors incubated with and without *Sporomusa ovata* at day 17 (experiments described in this study). In addition, results of a similar experiment, but with decreased headspace flushing, are included. The measurements were obtained either with GC or microsensors. The H_2 level measured in the gas phase (ppm) using GC was converted to the dissolved H_2 concentration using the Henry's law constant. For the microsensor measurement, the concentration measured at 5000 µm distance from the cathode was used (**Figure 3**). Dissolved H_2 concentrations are presented in µM.

**Dissolved H_2 concentration at gas-liquid interphase**		**Estimated from headspace /µM**	**Measured with microsensor /µM**
**Normal headspace flushing (experiment described in this study)**	abiotic	95.2	32.9
	S. ovata	0.2	1.5
**Decreased headspace flushing (similar experiment)**	abiotic	161.2	169.0
	S. ovata	0.1	2.7
